# Predicting Real-Life Eating Behaviours Using Single School Lunches in Adolescents

**DOI:** 10.3390/nu11030672

**Published:** 2019-03-20

**Authors:** Billy Langlet, Petter Fagerberg, Anastasios Delopoulos, Vasileios Papapanagiotou, Christos Diou, Christos Maramis, Nikolaos Maglaveras, Anna Anvret, Ioannis Ioakimidis

**Affiliations:** 1 Innovative Use of Mobile Phones to Promote Physical Activity and Nutrition across the Lifespan (the IMPACT) Research Group, Department of Biosciences and Nutrition, Karolinska Institutet, 14152 Stockholm, Sweden; petter.fagerberg@ki.se (P.F.); Ioannis.Ioakimidis@ki.se (I.I.); 2 Multimedia Understanding Group, School of Electrical and Computer Engineering, Aristotle University of Thessaloniki, 54124 Thessaloniki, Greece; adelo@eng.auth.gr (A.D.); vassilis@mug.ee.auth.gr (V.P.); diou@mug.ee.auth.gr (C.D.); 3 Laboratory of Medical Informatics, School of Medicine, Aristotle University of Thessaloniki, 54636 Thessaloniki, Greece; chmaramis@med.auth.gr (C.M.); nikolaos.maglaveras@northwestern.edu (N.M.); 4 Mandometer Clinics, 14104 Stockholm, Sweden; Anna.Anvret@mando.se

**Keywords:** Cohen’s kappa, sensory science, eating behaviour, nutrition, novel technology, overweight, meal duration, recording frequency, confidence interval, reliability

## Abstract

Large portion sizes and a high eating rate are associated with high energy intake and obesity. Most individuals maintain their food intake weight (g) and eating rate (g/min) rank in relation to their peers, despite food and environmental manipulations. Single meal measures may enable identification of “large portion eaters” and “fast eaters,” finding individuals at risk of developing obesity. The aim of this study was to predict real-life food intake weight and eating rate based on one school lunch. Twenty-four high-school students with a mean (±SD) age of 16.8 yr (±0.7) and body mass index of 21.9 (±4.1) were recruited, using no exclusion criteria. Food intake weight and eating rate was first self-rated (“Less,” “Average” or “More than peers”), then objectively recorded during one school lunch (absolute weight of consumed food in grams). Afterwards, subjects recorded as many main meals (breakfasts, lunches and dinners) as possible in real-life for a period of at least two weeks, using a Bluetooth connected weight scale and a smartphone application. On average participants recorded 18.9 (7.3) meals during the study. Real-life food intake weight was 327.4 g (±110.6), which was significantly lower (*p* = 0.027) than the single school lunch, at 367.4 g (±167.2). When the intra-class correlation of food weight intake between the objectively recorded real-life and school lunch meals was compared, the correlation was excellent (*R* = 0.91). Real-life eating rate was 33.5 g/min (±14.8), which was significantly higher (*p* = 0.010) than the single school lunch, at 27.7 g/min (±13.3). The intra-class correlation of the recorded eating rate between real-life and school lunch meals was very large (*R* = 0.74). The participants’ recorded food intake weights and eating rates were divided into terciles and compared between school lunches and real-life, with moderate or higher agreement (κ = 0.75 and κ = 0.54, respectively). In contrast, almost no agreement was observed between self-rated and real-life recorded rankings of food intake weight and eating rate (κ = 0.09 and κ = 0.08, respectively). The current study provides evidence that both food intake weight and eating rates per meal vary considerably in real-life per individual. However, based on these behaviours, most students can be correctly classified in regard to their peers based on single school lunches. In contrast, self-reported food intake weight and eating rate are poor predictors of real-life measures. Finally, based on the recorded individual variability of real-life food intake weight and eating rate, it is not advised to rank individuals based on single recordings collected in real-life settings.

## 1. Introduction

The “Ending Childhood Obesity” WHO committee report of 2016, identifies prevention and control of overweight and obesity as a core priority in combating the obesity epidemic. Specifically, the report recommends that researchers should investigate the effect eating behaviour has on weight gain [1].

In the laboratory, food properties, sensory experience and the environment are all factors which have been shown to alter eating behaviour parameters and in turn, short term energy intake [2,3,4]. Two of the eating behaviour parameters which are often associated with increased energy intake are high eating rates and large portion sizes [5,6]. A Cochrane review recently concluded that reducing portion size could potentially reduce daily energy intake between 8.5% and 13.5% [5]. Meanwhile, a systematic review showed how reducing the amount of food eaten per unit of time (eating rate, g/min) by verbal and electronic feedback and by manipulating food properties can significantly reduce energy intake [6]. In line with the laboratory studies, real-life studies have shown a lower prevalence of overweight and obesity in individuals with a low eating rate [7,8]. Additionally, training individuals to eat in a healthy way is a common suggestion for developing effective prevention and intervention strategies to combat overweight and obesity [9,10].

In addition, studies in the laboratory suggest that an individuals’ eating behaviour is stable relative to the group. In test-retest conditions the correlation coefficient of eating behaviour parameters is usually above 0.75, both for solid and semi-solid food [11,12,13,14,15]. Similarly, most individuals maintain their eating style when energy density, texture, unit size and serving occasion is manipulated [14,16]. In line with these results, laboratory research has also found that groups that rate themselves as “fast eaters” usually eat significantly faster than groups rating themselves as “slow eaters” [17,18]. The relative stability of eating behaviour parameters of individuals and the significant difference between groups, divided based on self-report measures, suggest that short term screening of either subjectively or objectively measured eating behaviours could be enough to capture an individual’s true mean eating behaviour value. However, a recent study found that on an individual level the agreement between subjectively and objectively measured eating rate was low [17].

Additionally, some researchers argue that laboratory settings often lack some of the strongest predictors of food selection and intake, such as monetary and physical cost of acquiring food, as well as restrictions imposed by social- and work schedules [19,20]. This may in turn result in laboratory models being poor representations of real-life eating behaviour. An example of such a discrepancy was found in a study where a group of self-rated high eating rate individuals displayed a significantly higher eating rate when eating lunch in the laboratory, compared to a group which reported lower eating rate, but showed no significant difference to the low eating rate group during a real-life breakfast [18]. More research is required to evaluate if short term screening of eating behaviour in controlled environments, like the laboratory, can be used to predict real-life eating behaviour and in the long run help identify individuals at risk of developing overweight and obesity.

Recent technological developments have resulted in smaller and more user-friendly devices, making it possible to objectively measure eating behaviour in real-life environments using, for example, personal food scales [21,22]. Exploiting this potential, our current study aimed to compare meal characteristics for individuals between: (i) single, semi-controlled meals of adolescents, monitored in a school cafeteria setting, against (ii) real-life meals from the same individuals, objectively recorded in a “hands-off” fashion for at least two weeks. Specifically, we hypothesised that the food intake weight and the eating rate of recorded semi-controlled meals would predict the average food intake weight and eating rate of adolescents in real-life, allowing us to correctly rank individuals (e.g., “fast” vs “slow” eaters) in comparison to their peers.

## 2. Materials and Methods

### 2.1. Subjects

Participants were recruited from a high school situated in central Stockholm. All students from six classes were informed of the study, which had no additional exclusion criteria. However, the recruitment rates for the study were also dependent on the availability of the deployed devices (26 food recording devices were available), thus the interested participants were included in the study on a “first-come first-serve” basis. Older participants (≥18 years) signed written consent, while younger participants signed written assent and written consent was provided by their legal guardians. The presented protocol was approved by the Stockholm Regional Ethics Board (D.nr.: 2015/1824-31 and 2016/598-31) and the presented practises follow the guidelines for human research in the Declaration of Helsinki. The compensation for participating in the study was three cinema tickets.

### 2.2. Experimental Design

During the study, the eating behaviour (food intake weight and eating rate) of the participants was quantified in two different settings. In the first setting, participants self-rated their food intake weight and eating rate, after which it was recorded objectively during a supervised school lunch. In the second setting, subjects participated in a data collection action lasting at least two weeks, during which they recorded as many main meals (i.e., breakfast, lunch and dinner) as they could in real-life. 

### 2.3. School Lunch Session

Each subject participated in one supervised school lunch session, during a school day, following their regular class schedule. The school lunch session started by measuring each participant’s height and weight using a stadiometer and weight scale, respectively. Afterwards, the participants self-rated their food intake weight (“How much do you eat compared to others”) and eating rate (“How fast do you eat compared to others”) on a 5-point rating scale. Food intake weight answers ranged from; 1—“Much less” to 5—“Much more,” while eating rate answers ranged from; 1—“Much slower” to 5—“Much faster.”

At the time of their regular school lunch, participants were seated at a random position in a dedicated part of the school cafeteria, having access to food from a nearby buffet station. The participants were free to take as much food and drinks as they wanted, were free to revisit the buffet as many times as they wished and did not have to finish all the food placed on their plates to end their meals. All the individual meals were recorded by personal food scales (Mandometer^®^, TEM, Istanbul, Turkey) placed under the plates of the students. After the meal, subjects went on with their regular school schedule. The described protocol is very similar to the usual school practices in the participating high school and it follows a protocol previously employed in this setting [21].

For all the meals, three main dishes were provided by the school catering company; one meat, one fish and one vegetarian option. During the school lunch sessions, the dishes served were beef patty, vegan patty and fish filet with a side serving of boiled potatoes and a brown or white sauce option. The beverage options were water and/or milk. Participants were also allowed to serve themselves from a salad buffet, with an assortment of vegetables, including; iceberg lettuce, tomatoes, cucumber, carrots and brown beans. The salad buffet also included a few food items that varied from day to day, depending on the food served, which during the experiment was cottage cheese and jam. Participants could mix the main dishes, the side servings and add sauce and salad on the same plate without constrains. The dedicated buffet station was located between 4 to 8 m away, depending on the seating position of each participant. The above-mentioned procedures were identical to regular school lunch practices, allowing the students to follow their everyday lunch habits in the school cafeteria.

### 2.4. Real-Life Session

The protocol for the real-life session was uncontrolled by design, since the aim was to capture every-day eating behaviour in a naturalistic setting. Participation in the real-life session consisted of at least two consecutive weeks of recordings. In total, participants had the possibility to record meals during, at least, 10 school days (Monday to Friday) and four weekend days (Saturday and Sunday). Participating students followed their normal school and out-of-school schedules, without any restrictions due to the experimental protocol.

Participants were instructed to record as many main meals (breakfast, lunch and dinner) as possible, using the provided mobile app and food scale. The maximum number of main meals that could be recorded with the application per day, per participant, was three (one breakfast, one lunch and one dinner). Thus, 21 recordings per week would be required for a participant to exhibit a 100% recording frequency.

### 2.5. Device and Mobile Application

The food scale used for both the school lunch and real-life session was a medical device called the Mandometer^®^ v5 [23]. The Mandometer^®^ is a portable food scale linked via Bluetooth^®^ to a smartphone, which provide continuous recording of food weight on the plate. The device is used by putting a plate on the scale and then adding food to the plate. The device records weight reduction over time at a sampling rate of 1 Hz.

The recording action was supported through a mobile application specifically designed for the study [24]. Using the app, the meal time and metadata (e.g., meal type) was available for each recorded meal. This data was uploaded through Wi-Fi online to a server, allowing the researchers to supervise the progress of the study in real time. Server side, an automatic algorithm calculated the food intake weight, meal duration and eating rate of all meals [25,26]. Finally, a researcher performed visual inspections on each algorithm-corrected recording, removing the ones that were not meals and correcting obvious errors based on the process described by Ioakimidis, et al. [27]. Parts of these features have already been used in a controlled *school lunch* setting, identical to the *school lunch* setting in the current study, with users reporting high acceptance of both the devices and the study protocol [21].

### 2.6. Data Analysis

The presented figures and statistical analyses were done using R 3.5.1 [28]. A power calculation was performed aimed at detecting a difference in food intake weight of 50 g between the *school lunch* meal and the mean value of real-life meals, with α = 0.05 and β = 0.80, using pilot data from a previous analysis of school-based lunches in a comparable population [21], identifying a minimum required sample size of 16. Shapiro–Wilk tests, Q-Q plots and residual vs fitted value plots were used to ensure the fulfilment of the normality assumption, both for the food intake weight and meal duration of the meals.

Group level comparisons of food intake weight and eating rate were performed using paired t-tests. For the description of the range of food intake weight and the eating rate within the meals of single individuals, all confidence intervals are unadjusted 95% CI. In order to assure a proper sampling per individual, this analysis included only participants with seven or more real-life recordings (8% excluded). For reliability measures and to evaluate potential familiarisation effects of the protocol, the first seven consecutive meal recordings from the real-life meals were compared in an iterative process (1 to 2, 2 to 3, 3 to 4, etc.) [29]. For clarity, in line with recommendations by Hopkins, the presented results of food intake weight and eating rate were based on logarithmically transformed data during the individual real-life variance analysis, although results from the untransformed data was similar [30]. In the results, intra-class correlations (ICC) and mean differences are displayed as percentages and standard error of measurement (SEM) is presented as a coefficient of variation (COV). The intra-class correlation thresholds were set at R; <0.10 (trivial), 0.1–0.3 (small), 0.3–0.5 (moderate), 0.5–0.7 (large), 0.7–0.9 (very large), 0.9–1.0 (excellent), respectively.

Since no participants selected the extreme food categories (“Much less” or “Much more”), these were omitted, creating an identical number of categories as in the paper by van den Boer [17]. Thus, only three self-rated food intake weight categories (“Less,” “Average” and “More”) were finally analysed. For self-rated eating rate only one participant rated eating “Much slower” and three rated themselves eating “Much faster” than their peers and were therefore merged with the “Slower” and “Faster” category, respectively. To enable comparison with the self-rated data, the objective recordings of food intake weight during the school lunch and real-life session were placed into three categories, corresponding to the lowest, intermediate and highest measurements in our sample. Thus, by design, eight measurements were included in each of the created ranks. The same procedure was employed for objective recordings of eating rate. These categories were then compared using Cohen’s kappa analysis, with agreement thresholds set at κ; <0.00 (less than chance), 0.01–0.20 (slight), 0.21–0.40 (fair), 0.41–0.60 (moderate), 0.61–0.80 (substantial) and 0.81–0.99 (almost perfect). All the values presented in the text are mean (SD), unless otherwise specified and all statistical tests are based on a significance threshold of 0.05.

## 3. Results

### 3.1. Subjects

A total of 26 adolescents participated in the study, which was the maximum amount possible due to the number of available food recording devices. Two female participants were excluded for not following the study protocol during the real-life measurement period, which resulted in 24 participants being included in the statistical analysis (Table 1).

### 3.2. Recording Frequency

**Figure 1:**
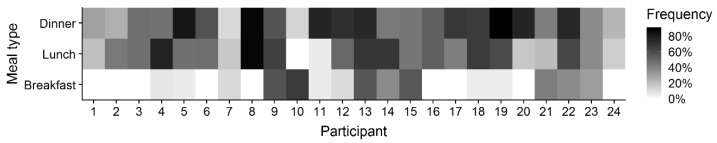
Tile plot depicting the recording frequency (0%–100%) of individual meal types (breakfast, lunch and dinner) per participant, based on the maximum potential number of meal type recordings during the study.

Over the course of the study, participants recorded on average 18.9 (7.3) main meals, which equals 34% of the theoretical maximum number of meals expected in the case of all participants recording one breakfast, lunch and dinner each day. The mean number of breakfast recordings was 2.8 (3.6), while the mean number of lunch and dinner recordings were 7.2 (3.8) and 8.9 (4.3), respectively. During the first week, the participants recorded 32% of their potential main meals, while 39% of potential main meals were recorded during the second week. On an individual level the number of main meal recordings ranged from 5 to 31, with all subjects recording at least two dinner meals, all but one subject recording at least one lunch and 15 subjects recording at least one breakfast, over the course of the study (Figure 1).

### 3.3. Real-Life Variance

**Figure 2:**
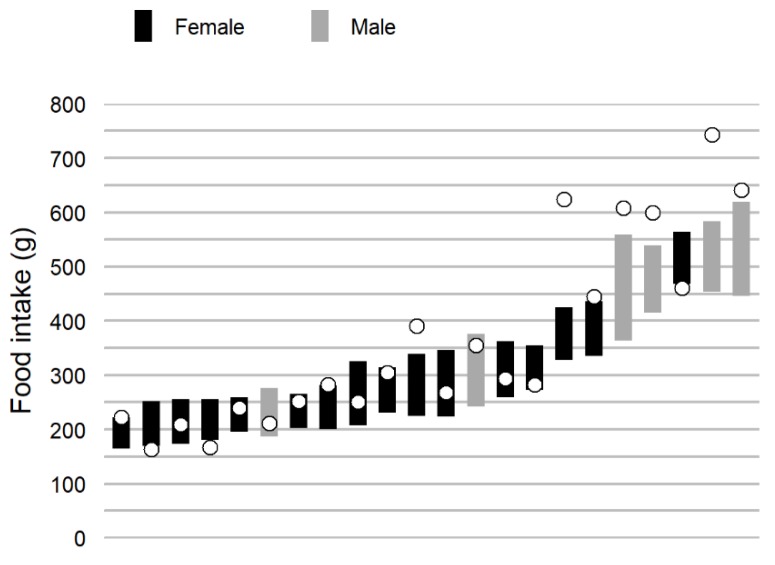
Confidence intervals for food intake weight of each participant, arranged in ascending order. Based on all real-life meal recordings, only including participants with ≥7 recordings. The white dots represent the single school lunch recording of each participant.

The food intake weight difference between consecutive meals from the first to seventh measurement resulted in a mean difference from −9.0% to 5.6%, a SEM (expressed as a coefficient of variation) from 47.5% to 50.9% and a correlation coefficient between 0.03 and 0.49. Neither the mean difference, standard deviation nor correlation displayed a trend across measurements, with later measurements not systematically differing compared to earlier ones. The confidence interval of food intake weight derived from all meals recorded during the *real-life* session resulted in individual intervals from 23.7 to 78.5 g, with a mean confidence interval of 47.9 g (Figure 2).

The eating rate difference between consecutive meals from the first to seventh measurement resulted in a mean difference from −12.6% to 24.4%, a SEM from 36.2% to 55.4% and a correlation coefficient between 0.55 and 0.74. Neither the mean difference, standard deviation nor correlation displayed a trend. The confidence interval of eating rate derived from all meals recorded during the *real-life* session resulted in individual confidence intervals between 3.1 and 8.6 g/min, with a mean confidence interval of 5.4 g/min.

### 3.4. Comparison of School Lunch and Real-Life Food Intake Weight and Eating Rate

**Figure 3:**
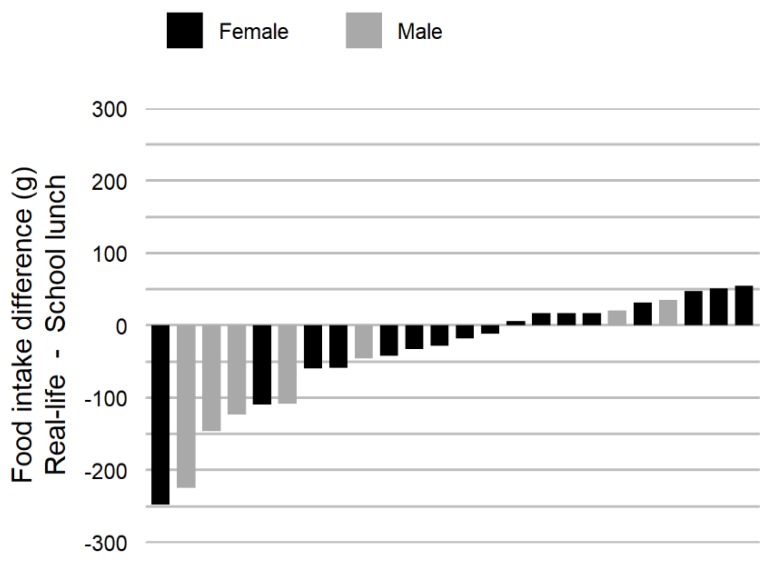
Food intake weight difference between the school lunch and the mean value of all real-life meals.

Food intake weight during the school lunch was significantly higher compared to food intake weight at the real-life meals, while the correlation was excellent between the two conditions (Table 2). On an individual level, the food intake weight difference between the school lunch and the mean of all recorded real-life meals ranged from −247.6 to 54.6 g (Figure 3). Food intake weight data resulted in a mean difference of 7.9% and a SEM of 14.1%, between the school lunch and real-life meals.

There was a significantly lower eating rate during the school lunch compared to the real-life meals, while the correlation was very large between the two conditions (**Table 2**). On an individual level, the eating rate difference between the school lunch and real-life meals ranged from −5.5 to 55.0 g/min. Log transformed eating rate data resulted in a mean difference of −16.2% and a SEM of 35.9%, between the school lunch and real-life meals.

### 3.5. Agreement between Measures

Agreement between the terciles (“Less”/”Slower,” “Average” and “More”/”Faster”) of the *real-life* and *school lunch* food intake weight and eating rate was κ = 0.75 (*p* > 0.001) and κ = 0.54 (*p* > 0.001), respectively. A similar approach, comparing the *real-life* food intake weight categories to the self-rated food intake weight and eating rate resulted in Cohen kappa values of κ = 0.09 (*p* = 0.469) and κ = 0.08 (*p* = 0.509), respectively.

## 4. Discussion

This is the first study objectively evaluating food intake weight and eating rate stability of real-life meals and exploring the potential to use single meal measures of food intake weight and eating rate in a supervised school lunch to predict real-life mean values, at group and individual level. Since both measures have previously been implicated as risk-behaviours associated with the development and maintenance of obesity, our current effort has the potential to enhance the effectiveness of prevention strategies aiming at tackling the obesity epidemic.

On a group level, considering only real-life measures, the mean difference of food intake weight between consecutive real-life recordings was small (less than ±10%), while eating rate was larger (±25%), pointing towards higher group stability for food intake weight measures, and less so for eating rate measures. With no apparent trend in mean difference, nor standard deviation or correlation during consecutive recordings our protocol did not appear to have a habituation or familiarisation effect on food intake weight or eating rate. The large individual variance and the low correlation between consecutive meals suggests a low reliability of single real-life meal recordings. Therefore, it would not be advised to rank individuals based on a single real-life recording. 

On an individual level, the large difference in confidence interval ranges across individuals of both food intake weight and eating rate, suggests measuring manipulation/treatment response in real-life requires more measurements in certain individuals. It has been previously reported that meal-specific food intake is affected by different environmental parameters, just like food proximity [31] and accessibility [32], social environment [33]. In other cases, restricted time during the meal [34] has been shown to affect both eating rate and food intake weight in semi-controlled meals. All these parameters were, by design, not controlled in the present study. Past evidence also shows that certain individuals are more responsive to certain external cues and as such at higher risk of developing obesity in obesogenic environments [35], potentially contributing to the observed variation of food intake weight and eating rates within meals of the same subjects. There are a few studies that have investigated the variation of food intake weight across days [36,37], and while most of these studies rely on self-reports, it is interesting to note that one study found that estimating food energy intake within 10% required on average 31 recordings [38]. Currently no study has investigated the reliability of food intake weight and eating rate in single real-life meals and while there are a few that have used objective methods to investigate reliability in the laboratory, they do not report individual differences between measurements [11,12,13]. However, employing similar analysis methods in a study comparing lunch and dinner meals of slightly older female participants in the laboratory resulted in a mean change in food intake weight of 5 g (2%) and a SEM of 16% and a mean change in eating rate of 0.14 g/min (−0.2%) and a SEM of 11%, which is more than three times lower than the variance in the current study [16]. As expected, this comparison suggests that the measurement error is increased when moving from the laboratory to real-life.

Regarding the recording frequency in the real-life session, the employed study protocol and technology enabled, on average, 19 recordings per individual over a course of at least two weeks. However, the low number of recordings during breakfast resulted in only 63% of subjects having at least one recorded real-life measurement. This means both group and individual results may be more representative of lunch and dinner, than breakfast. The reason for this discrepancy is unknown but could stem from social desirability (e.g., where individuals only record meals/products that are viewed favourably by others) [39] food being eaten on the go, or some breakfasts not being suitable for recording using the device. The increased number of recorded main meals from week one to week two suggest that a familiarisation period, or more training in device use can increase recording frequency. One way of removing the potential training period and reporting bias would be to employ automatic recording methods, such as the ones developed by Sazonov et al. [40], Sun et al. [41], and Kyritsis et al. [42]. However, these methods require validation to ensure the underlying assumptions of the algorithms do not introduce other biases to the measures.

The significant difference in food intake weight and eating rate between the controlled school lunch and real-life meals strengthens the assertion that eating behaviour in controlled settings is different to real-life [19,20]. However, the high correlation of both food intake weight and eating rate indicates that most individuals maintain their eating behaviour, in relation to the group, between settings. That is to say, a “fast eater” in controlled conditions, is likely a “fast eater” in real-life as well. This corroborates the findings of laboratory studies, which often show a correlation coefficient >0.75 of food intake weight and eating rate for identical foods, as well as foods of different energy density, texture and unit size [11,12,13,14,16].

The agreement between self-rated and real-life food intake weight and eating rate were both close to what would have been expected by chance, suggesting self-reported food intake weight and eating rate are poor predictors of real-life measures. These results are in part corroborated by findings by van den Boer et al. [17], conducted in a laboratory setting on young adults, where they evaluated the agreement between self-rated and objectively measured eating rate. It is also in line with findings by Petty et al., comparing the eating rate of a laboratory lunch to a real-life breakfast, where they found that the group with a significantly higher eating rate during the laboratory lunch did not differ significantly to the low eating rate group during the real-life breakfast [18]. However, there was a high agreement of food intake and, to a slightly lesser extent, eating rate between the *school lunch* and *real-life* setting. This suggests that, in groups like the current sample, controlled *school lunches* perform far better than subjective ratings to divide individuals into eating style categories, such as “high food intake” and “low food intake” and could potentially be used to identify groups at risk of developing obesity, assuming these eating behaviours are strong mediators.

The main strengths of the study were; (i) a device which allowed high measurement accuracy of food intake weight and eating rate, (ii) an app designed to allow easy real-life recording and (iii) a controlled setup during the school lunch session, while remaining similar to regular school practices. The homogenous sample is considered a strength in this study since less homogenous samples may increase correlation coefficients merely as a result of high group variance. Additionally, a study conducted a year earlier in the same school lunch setting produced a similar food intake weight value [21], making us confident that the study procedures and environment was similar to the previous study. 

The main limitation of the study was a comparatively low number of participants. However, the number of subjects included in the statistical analysis was still larger than required, based on the power calculation, despite the limited number of available devices. The low number of male participants is another weakness of the sampling process, but this was expected since more women attend high-school in Sweden, compared to men. Another potential limitation was the inability of the app to quantify serving size, since there appears to be a difference between serving size and food intake weight in response to factors such as serving situations (serving oneself vs. being served by others) and food types (main meal vs. snack) [43]. In the current study, only 19% of participant were either overweight or obese, which is within the confidence interval of WHOs report on childhood obesity in Sweden [44]. However, it is in the lower end and one should be careful to make to broad generalizations based on this data. Another potential limitation is the three main meals per day assumption for compliance estimation, since meal-skipping studies suggest that not everyone eats breakfast, lunch and dinner [45]. It is also important to note that, while some laboratory studies are conducted in settings like the one used in this study (school lunch), most laboratory studies control more potential variables (e.g., removing all dining companions and side servings). Finally, we need to emphasize that the recorded meal measurements are not equivalent with energy intake per meal, since the current protocol did not allow us to quantify the energy density of the selected foods.

Future studies should aim to recruit enough obese individuals to evaluate the risk posed by a high food intake weight and eating rate. Future studies should also collect even more recordings per participant, both in supervised school lunches and in real-life settings. They should also try to collect multiple recordings per individual in even more controlled settings, such as traditional microstructural recordings in the laboratory, to determine the generalisability of various laboratory protocols. For example, ad libitum laboratory studies [16] may be comparable to real-life buffet settings, while preload studies [46] may be comparable to full-course restaurant meals. Recording more meals per participant in the supervised school lunch would allow the detection of potential outlier meals and provide an estimate of the measurement error. In the real-life setting more recordings, aided by photos and/or annotations, would enable the identification of eating behaviours differences between main meals (e.g., lunch vs. dinner), environments (e.g., restaurant vs. home dining) and food properties (e.g., liquid vs. solid food), on an individual level. In addition, validation studies should be performed between the current method (real time report and record) and automatic recording alternatives (e.g., eButton and Automatic Ingestion Monitor) [40,41]. Future studies should also aim to improve the study protocol to enable the quantification of serving size of meals, which may differ from food intake weight [47] and the recording of snacks, where consumption patterns may differ between groups (e.g., between normal weight and obese individuals) [48]. Another improvement would be the automatic identification of the selected food types, through for example picture analysis [49] and potentially energy density of each recorded meal. Additionally, as more sensors become available to quantify various parts of human eating behaviour, it becomes increasingly interesting to use them together to reduce the number of inferential measures and provide a more detailed account of the food properties and environments of each meal. Exploring the strengths and limitations of new technologies will enable the quantification of human behaviour in more relevant environments and is expected to improve the accuracy of measurements in environments where other methods for measuring human behaviour are already employed. The end goal of these type of studies is to provide valid prediction and response measures, enabling the development and evaluation of prevention and intervention strategies.

## 5. Conclusions

With the current sample size (*n* = 24), food intake weight values from single meal recordings in real-life produced more accurate group means than eating rate, which likely requires a larger sample size. The large differences in the confidence interval ranges of food intake weight and eating rate across individuals suggests that some individuals may have less stable eating patterns or be more responsive to environmental cues than others. The current study provides evidence that there is a difference between food intake weight and eating rate of controlled school meals and meals in real-life. It also suggests that one controlled school lunch is enough to rank an individuals’ real-life food intake with high accuracy, in relation to the group. Meanwhile, the accuracy is slightly lower when ranking an individuals’ real-life eating rate. In contrast, self-rated food intake weight and eating rate seem to be poor predictors of real-life food intake and eating rate. Finally, this study provides evidence that real-life recordings vary greatly within the individual and, as such, it is not advised to rank individuals based on single recordings collected in real-life settings.

## Figures and Tables

**Table 1:** Group characteristics.

	Total (*n* = 24)	Male (*n* = 7)	Female (*n* = 17)
Age, y	16.8 (0.7)	17.2 (0.5)	16.6 (0.7)
Weight, kg	62.2 (14.4)	75.7 (10.1)	56.6 (12.1)
Height, cm	168.1 (10.3)	181.3 (6.8)	162.7 (5.3)
BMI, kg/m^2^	21.9 (4.1)	23.3 (4.7)	21.3 (3.8)

Values expressed as mean (SD).

**Table 2:** Mean (SD) values of food intake weight and eating rate of recorded meals.

	Real-Life ^m^	School Lunch	Diff. ± 95% CI	*p*-Value	Correlation
Food intake weight, g	327.4 (110.6)	367.4 (167.2)	−40.0 ± 35	0.027 *	0.92
Eating rate, g/min	33.5 (14.8)	27.7 (13.3)	5.8 ± 4.3	0.010 *	0.75

^m^ Real-life includes all meals recorded during the *real-life* session, where the group mean (SD) values were derived from the mean value of all meals of each participant. * *p* < 0.05.
